# Breast myeloid sarcoma presenting as a palpable breast lump after allogeneic stem cell transplantation for acute myelomonocytic leukemia: a rare case report

**DOI:** 10.1186/s12957-021-02399-9

**Published:** 2021-09-27

**Authors:** Chengmin Huang, Shengqi Fei, Jiang Yao, Panpan Chen, Jiaqing Luo, Yaqi Wang, Jie Li, Weilan Wang

**Affiliations:** 1 Department of Surgery, Changxing People’s Hospital, No. 66, Taihu Road, Changxing, Huzhou, 313100 China; 2 Department of Hematology, The Second Affiliated Hospital, College of Medicine, Zhejiang University, Hangzhou, 310009 China

**Keywords:** Breast myeloid sarcoma, Acute myeloid leukemia, Stem cell transplantation, Extramedullary manifestation, Case report

## Abstract

**Background:**

Myeloid sarcoma (MS) is a tumor secondary to myeloid leukemia that consists of immature granulocytes with or without mature granulocytes and is a rare extramedullary manifestation of acute myeloid leukemia (AML).

**Case presentation:**

We report a case of a 34-year-old woman diagnosed with AML-M4 who achieved remission after chemotherapy and received allogeneic stem cell transplantation (allo-SCT) for consolidation. Her past medical history showed that she received bilateral breast implants 7 years ago. This patient underwent ultrasound examination of the breast and multiple bilateral breast nodules were revealed that were not considered by clinicians to be concerning. Several months later, the patient’s bilateral nodules rapidly progressed to large palpable masses. Ultrasound-guided biopsy revealed diffuse infiltration of undifferentiated tumor cells and immunohistochemistry (IHC) indicated that the tumor was positive for myeloperoxidase (MPO), cluster of differentiation (CD) 34, CD43, CD68, CD117, and Ki67. The pathological diagnosis was extramedullary recurrence of AML as MS of breast. After the diagnosis, the patient received systemic chemotherapy and drugs containing cytarabine, azacitidine, and methotrexate. However, 1 year after achieving partial remission, the patient died from intracranial invasion of leukemia, brain herniation, and respiratory failure.

**Conclusion:**

It is necessary for the specialist to have a high suspicion index by careful inquiry of the patient’s medical history if a patient presents at the breast clinic with a breast tumor as the chief complaint. Combining information from the patient’s medical history with a tumor biopsy is critical for obtaining the correct diagnosis of the disease.

## Background

Myeloid sarcoma (MS), also known as granulocytic sarcoma (GS) or chloroma, is a rare extramedullary manifestation of acute myeloid leukemia (AML). It is defined as the extramedullary proliferation of one or more myeloid cells that destroys the normal structure of the tissue. MS can occur at any age and site. The most common organs involved include the skin, lymph nodes, genitals, gastrointestinal tract, bones, and central nervous system [1–5]. Although the majority of cases arise in patients with AML, the disease may rarely manifest without or prior to medullary involvement. Breast MS is very rare and only accounts for approximately 8% of cases [6]. Despite the rarity of the disease, this tumor should not be overlooked [7]. Extramedullary relapse can be seen in 5–12% of patients after allogeneic stem cell transplantation (allo-SCT) [8–10]. The clinical presentation is nonspecific, and the diagnosis is challenging, especially in patients with primary breast involvement and no evidence of medullary disease [11, 12]. Despite the importance of breast imaging for the initial assessment of the disease, this technique lacks specificity, and MS can mimic several other tumors, including breast carcinoma and lymphoma. Moreover, only a few reports on breast MS have been published to date [13–16]. Currently, only histological examination with IHC can be used to confirm the diagnosis of breast MS [7, 17]. Here, we report the case of a patient presenting with multifocal MS of the bilateral breast after several months of confirmed AML remission with special focus on sonographic and MRI examination.

## Case presentation

Here, we present the case of a 34-year-old female patient who was admitted to our Hematology and Bone Marrow Transplantation Center in December 2018. She had malaise, was febrile for 1 week, had no purpura or petechiae, and had no enlargement of the liver or spleen. A complete blood count (CBC) showed leukocytosis (6927/μL), anemia (hemoglobin 4.8 g/dL), and thrombocytopenia (2000/μL). In the peripheral blood smear, 56% of the cells were blasts. Bone marrow aspirate showed an excess of bone marrow primitive cells, 76% of which were blast cells, suggesting an AML myelogram. Immunophenotyping by flow cytometry revealed two leukocyte populations. A total of 68.4% of the bone marrow blast cells expressed CD13, CD117, and MPO, and some expressed HLA-DR, CD4, CD15, CD33, CD34, CD38, and CD64 and without CD61 expression. The final diagnosis was AML-M4 according to the French-American-British (FAB) AML classification, with no other description (NOS). Conventional karyotype analysis was performed on 20 metaphases, revealing that one cell had a normal karyotype (46, XX) and 19 cells had one abnormal alteration in which chromosome 16 had an interarm inversion, 46, XX, inv (16) (p13.1q22). Molecular biology testing was positive for the AML fusion gene CBFβ/MYH11 and other relevant AML genetic testing were negative including NPM1 and FLT3. According to AML risk stratification based on 2017 European LeukmiaNet (ELN), this patient is in the moderate-risk group. Then, AML induction “7+3” therapy (7 days of cytarabine (Ara-C) + 3 days of idarubicin (4-demethoxydaunorubicin)) was started, and partial remission (PR) was achieved because of minimal residual disease (MRD) of bone marrow aspiration was positive. Subsequently, the patient received one HA regimen (homoharringtonine and Ara-c) and one FLAG regimen (Flu + Ara-C + G-CSF) but still did not achieve complete remission. In July 2019, semicompatible allogeneic hematopoietic stem cell transplantation (HSCT) was performed with cells from the patient’s mother, preceded by a demyeloablative regimen of BuCy (leucovorin 180 mg d-7~-5, fodarabine 50 mg d-4~-2, cyclophosphamide 1950 mg d-3~-2, anti-human T lymphocyte globulin 150 mg d-4~d-1).

In January 2020, the patient noticed multiple painless and palpable masses located in multiple quadrants of her bilateral breasts. Clinical examination showed multiple painless masses, of which one lesion was located in the left breast measuring 3.0 cm × 1.2 cm, and a large mass of approximately 2.3 cm × 1.64 cm was palpable in the right breast without enlarged lymph nodes in her axilla. Ultrasound showed multiple nodules in both breasts. The tumors were assessed as BI-RADS-3 and were not considered to be a concern by clinicians.

In March 2020, only 8 months after allogeneic HSCT, the masses in the patient’s bilateral breasts developed rapidly and became large palpable tumors. The rapid growth of the tumors attracted the attention of the outpatient breast surgeon and the patient was admitted to the hospital. Laboratory tests revealed only a slight decrease in the number of white blood cells. Mammography was not performed because of discomfort caused by the patient’s breast implants. No AML cells were found in the bone marrow after a bone marrow puncture.

**Fig. 1:**
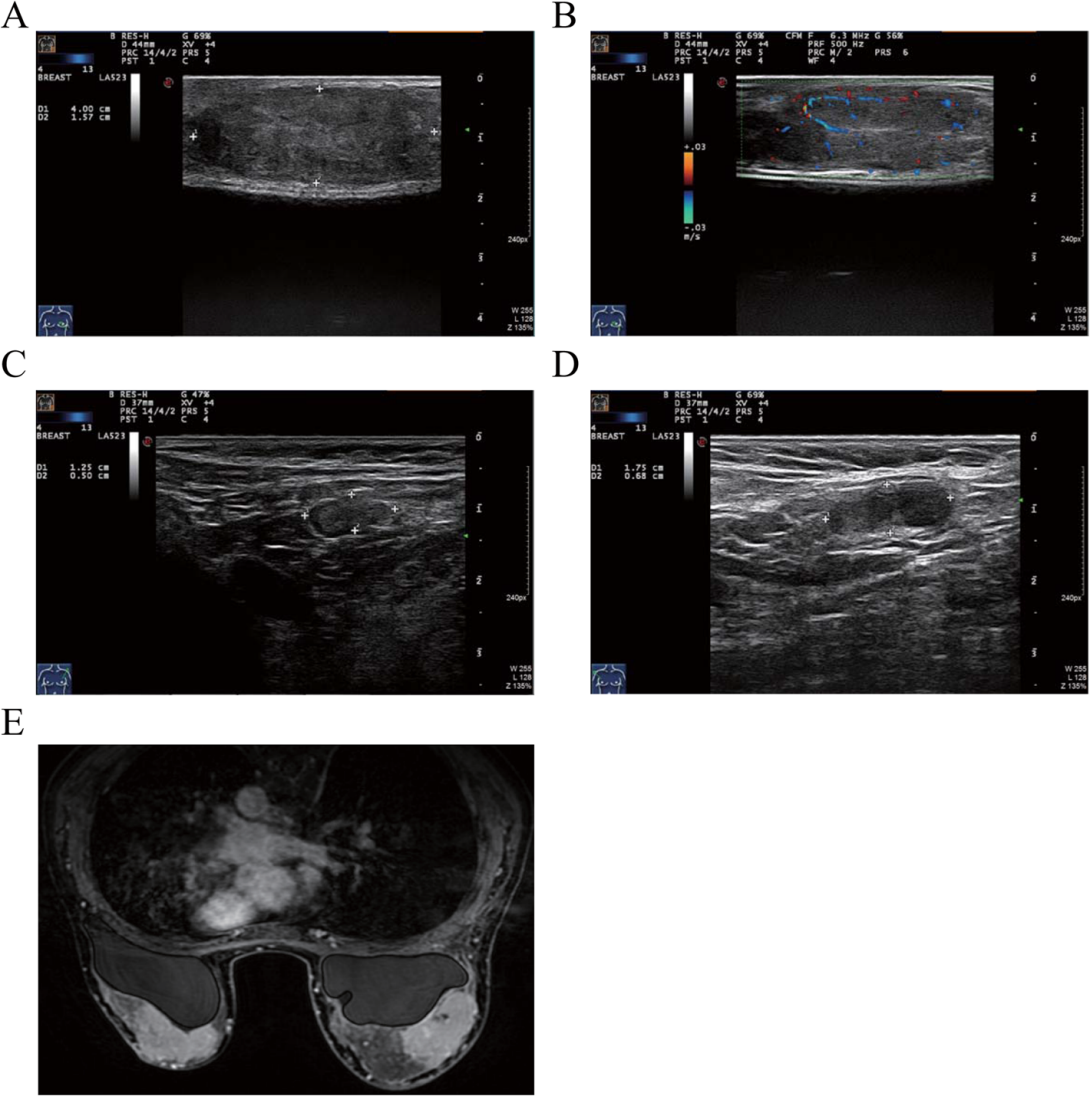
The ultrasound and MRI results of the breast masses. **A** Breast ultrasonography revealed masses with well-defined hypoechoic lesions in the left breast. **B** Masses with well-defined hypoechoic lesions were found in the right breast. **C** Hypoechoic nodules were seen in the left axillae (1.25 cm × 0.50 cm). **D** Hypoechoic nodules were seen in the right axillae (1.75 cm × 0.68 cm). **E** MRI showed hypointensity on T1-weighted images and hyperintensity on T2-weighted images

An ultrasound revealed multiple similar well-defined hypoechoic oval lesions that were classified as BI-RADS-4a scattered throughout the breast tissue. One of the largest breast lesions was located in the left breast, measuring approximately 3.09 cm × 1.64 cm, and the smaller lesion was approximately 3.04 cm × 2.28 cm in the right breast and corresponded to the known palpable mass. In April 2020, the patient underwent a second breast ultrasound; a 4.00 cm × 1.57 cm mass was found in the left breast (Fig. 1A) and a 4.40 cm × 1.17 cm mass was found in the right breast (Fig. 1B). Additionally, hypoechoic nodules were seen in both axillae (Fig. 1C and D). A breast MRI-enhanced scan showed that all lesions were hypointense on T1-weighted images and hyperintense on T2-weighted images. After injection of gadolinium-based contrast medium, most lesions demonstrated striking enhancement and measured approximately 3-4 cm. On the other hand, the palpable bilateral breast lesions presented as enhanced masses with small patches of fluid necrosis observed in the left lesions (Fig. 1E).

**Fig. 2:**
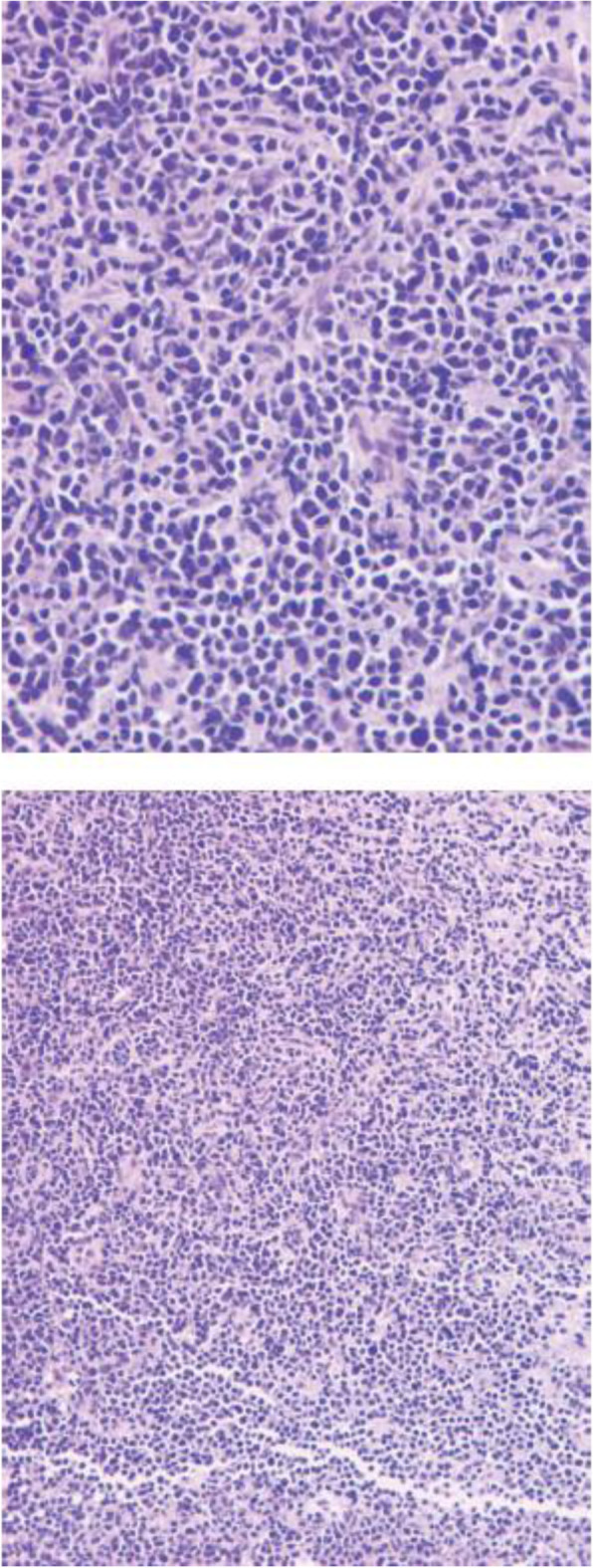
Breast mass biopsy. Microscopic examination of HE-stained slides revealed that the breast tissue was diffusely infiltrated with medium-sized malignant cells with round follicular nuclei, finely dispersed chromatin and small nucleoli, and minimal cytoplasm. **A** ×20, **B** ×40. HE, hematoxylin–eosin

**Fig. 3:**
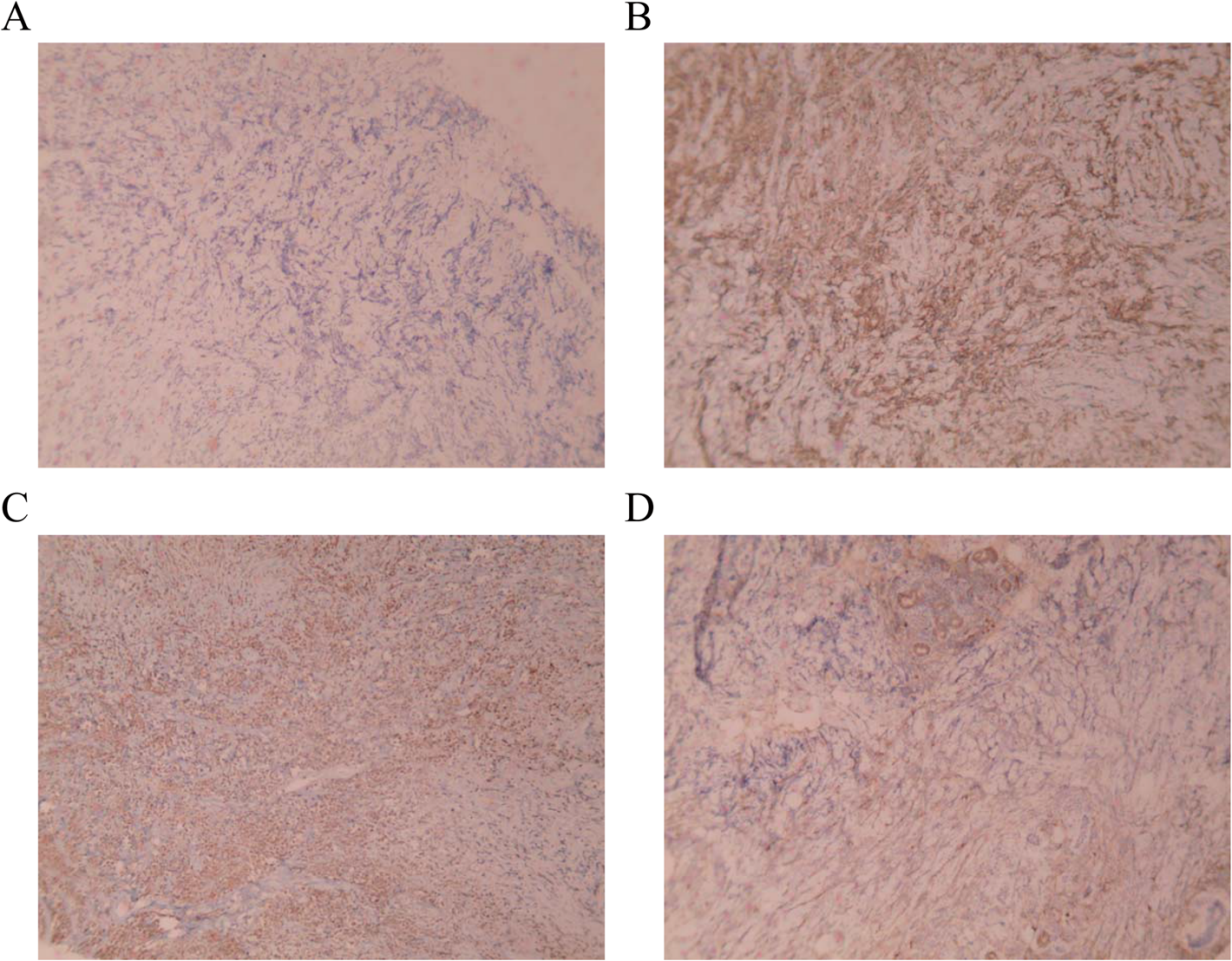
The IHC results of breast tumors. **A** Immunostaining for anti-CD34 (×100). **B** Immunostaining for anti-CD43 (×100). **C** Immunostaining for anti-MPO (×100). **D** Immunostaining for anti-CD117 (×100)

An ultrasound-guided needle biopsy was performed. Microscopic examination of HE-stained slides revealed that the breast tissue was diffusely infiltrated with medium-sized malignant cells with round follicular nuclei, finely dispersed chromatin, small nucleoli, and minimal cytoplasm (Fig. 2A and B). The IHC revealed that the tumors were positive for myeloperoxidase (MPO++), CD34 (+++), CD43 (+++), CD68 (+), CD117 (+), and Ki67 (40%+), which are markers of myeloid tumors (Fig. 3A-D). Molecular biology analysis was positive for the FLTD3-ITD mutation. Histological features were consistent with extramedullary AML of both breasts. The final pathological diagnosis was MS due to extramyeloid recurrence of leukemia.

**Fig. 4:**
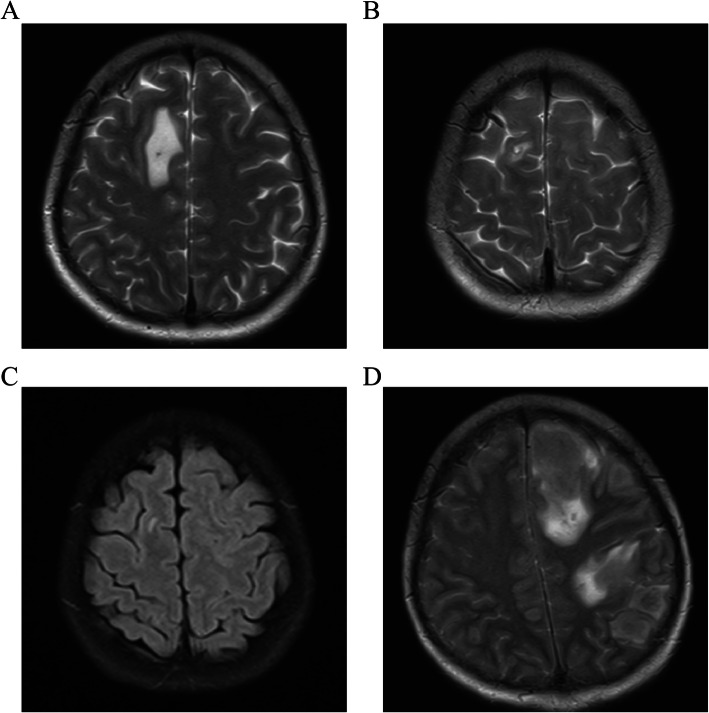
Head MRI for cranial occupancy. **A** The first cranial MRI. **B** The second cranial MRI. **C** The third cranial MRI. **D** The final cranial MRI

**Fig. 5:**
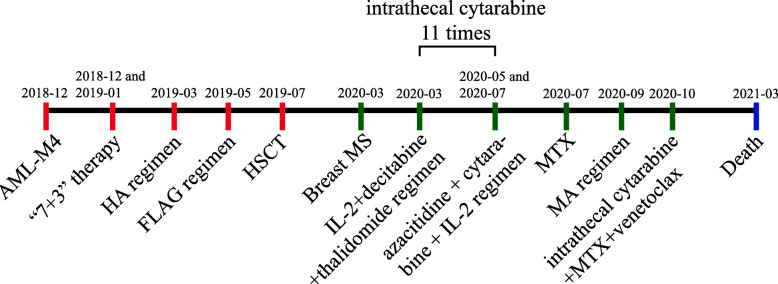
The timeframe regarding the treatment of the disease. AML-M4, acute myelomonocytic leukemia, M4 type; HA, homoharringtonine and Ara-c; FLAG, Flu + Ara-C + G-CSF; HSCT, hematopoietic stem cell transplantation; MS, myeloid sarcoma; IL-2, interleukin-2; MTX, methotrexate

Chemotherapy was resumed, and the patient received the interleukin-2 (IL-2) + decitabine + thalidomide regimen once, the azacitidine + cytarabine + IL-2 regimen twice, the methotrexate (MTX) regimen once, the MA (MTX + cytarabine) regimen once, and 11 doses of 50 mg intrathecal cytarabine. It happened to a certain extent of the skin rejection; however, basically disappeared after the treatment of dexamethasone. MR imaging showed that the patient had significantly smaller breast masses and a significantly smaller cranial occupancy than before (Fig. 4A), with a new signal in the left frontal lobe and corpus callosum in October 2020 (Fig. 4B). MTX + venetoclax chemotherapy was given again in combination with intrathecal injections of cytarabine. The patient had a slight reduction in occupancy (Fig. 4C) in December 2020. On March 27, 2021, the patient presented at the emergency room with lethargy. A head MRI scan showed that the patient had multiple lesions in the brain parenchyma on both sides, suggesting AML invasion (Fig. 4D). On March 29, 2021, the patient died of intracranial invasion of AML, brain herniation, and respiratory failure. In order to show the patient’s disease treatment process more clearly, we drew a timeframe regarding the treatment of the disease (Fig. 5).

## Discussion and conclusions

Granulocytic sarcoma is also known as myeloid sarcoma or chloroma. It is a rare extramedullary tumor composed of malignant immature cells and associated mainly with AML. It is a rare lesion in AML patients, in particular after allo-HSCT, and is even more rare in the absence of bone marrow involvement. According to a retrospective analysis of European Bone Marrow Transplantation (EBMT) patients, less than 1% of patients treated with allo-HSCT develop MS 4-56 months after HSCT.

The manifestation of myeloid sarcoma in the breast is rare. One study from Viadana et al. [2] revealed that out of 503 patients with leukemia on whom autopsies were performed; only 4 of the 235 AML patients had involvement of the breast [18]. Recently, Bubulac et al. [19] reported a 30-year-old female diagnosed with AML-M4 who achieved complete remission after chemotherapy and subsequently underwent allo-SCT for consolidation. After 5 months, the patient presented with right breast tumors resulting from AML relapse with breast MS. Another recent study [13] reported MS of the breast as a relapse of AML after stem-cell transplantation. In 2018, Cheng et al. [20] presented the case of a 46-year-old woman with a known history of AML who had bilateral breast masses with pain and itchiness. Pathology showed mononuclear cells, suggestive of breast leukemic infiltration. However, there were no similar case reports in 2020 and 2021 until now.

The clinical features of breast GS are nonspecific and may mimic primary breast cancer, and diagnosis is challenging for clinicians [7]. Breast MS usually presents as palpable, painful, or painless breast nodules involving one or both breasts. Moreover, imaging findings are also nonspecific. There are few descriptions of mammograms in the literature and no specific imaging findings have been outlined.

On ultrasound, breast MS manifest as homogeneous hypoechoic lesions that are hypervascularized on color Doppler scans cannot be easily distinguished from benign lesions. Generally, lesions are depicted as hypoechoic with microlobulations or spiculated margins. MRI is a valuable diagnostic tool, especially for patients with breast implants, dense glandular tissue, or pregnancy. In our case, the patient had a history of breast prosthesis implantation and was suitable for MRI examination. Generally, breast lesions show hypointensity on T1-weighted images and hyperintensity on T2-weighted images [21]. Antidiastole depends largely on the age of the patient and on their medical history. Based on physical examination and imaging, younger patients tend to present with benign breast masses, such as fibroadenomas and fibrocystic changes. Less common benign lesions include papilloma, hemangioma, and intramammary lymph nodes. More rarely, malignant lesions such as lymphoma and soft tissue sarcomas may be found, but those findings are even rarer than primary breast carcinoma in this age population [22]. In older women, the most important differential to consider remains primary breast carcinoma, especially multicentric breast carcinoma. Additionally, breast lymphoma and benign lesions must be considered [12].

In recent decades, the number of women undergoing breast augmentation has increased. Whether there is relation between breast implants and cancer, it remains unclear. Noels et al. [23] performed a meta-analysis of cohort studies including seventeen studies representing 7 cohorts. They found that women who have undergone cosmetic breast implantation do not have an increased risk of breast cancer. In one review [24], it showed that breast implants are not associated with an increased risk of breast cancer incidence or death. Brinton et al. [25] also suggested that there is no convincing evidence that breast implants alter the risk of nonbreast malignancies. It is worth noting that cosmetic breast augmentation adversely affects the survival of women who are subsequently diagnosed as having breast cancer [26]. Therefore, women who have undergone cosmetic breast implantation do not have an increased risk of breast cancer; however, it affects the survival of who are subsequently diagnosed as breast cancer.

If a patient presents at the breast clinic with a breast tumor as the chief complaint, it is necessary for the specialist to have a low suspicion index by careful inquiry of the patient’s medical history. Combining information from the patient’s medical history with a tumor biopsy is critical for obtaining the correct diagnosis of the disease. IHC staining, including for myeloid antibodies (CD34, MPO, CD117, and CD33), are essential for an accurate diagnosis of breast MS.

## Data Availability

All data during the study are included within the article.

## Abbreviations

MS: Myeloid sarcoma
AML: Acute myeloid leukemia
allo-SCT: Allogeneic stem cell transplantation
IHC: Immunohistochemistry
MPO: Myeloperoxidase
CD: Cluster of differentiation
GS: Granulocytic sarcoma
CBC: Complete blood count
FAB: French-American-British
PR: Partial remission
MTX: Methotrexate
EBMT: European Bone Marrow Transplantation
